# MicroRNA-29b Modulates Innate and Antigen-Specific Immune Responses in Mouse Models of Autoimmunity

**DOI:** 10.1371/journal.pone.0106153

**Published:** 2014-09-09

**Authors:** Apolline Salama, Nolwenn Fichou, Marie Allard, Laurence Dubreil, Laurence De Beaurepaire, Alexis Viel, Dominique Jégou, Steffi Bösch, Jean-Marie Bach

**Affiliations:** 1 LUNAM Université, Oniris, EA 4644 IECM, Nantes, France; 2 INRA, Nantes, France; 3 LUNAM Université, Oniris, UMR_A0703 PanTHER, Nantes, France; Ajou University, Republic of Korea

## Abstract

In addition to important regulatory roles in gene expression through RNA interference, it has recently been shown that microRNAs display immune stimulatory effects through direct interaction with receptors of innate immunity of the Toll-like receptor family, aggravating neuronal damage and tumour growth. Yet no evidence exists on consequences of microRNA immune stimulatory actions in the context of an autoimmune disease. Using microRNA analogues, we here show that pancreatic beta cell-derived microRNA sequences induce pro-inflammatory (TNFa, IFNa, IL-12, IL-6) or suppressive (IL-10) cytokine secretion by primary mouse dendritic cells in a sequence-dependent manner. For miR-29b, immune stimulation in RAW264.7 macrophages involved the endosomal Toll-like receptor-7, independently of the canonical RNA interference pathway. In vivo, the systemic delivery of miR-29b activates CD11b+B220− myeloid and CD11b-B220+ plasmacytoid dendritic cells and induces IFNa, TNFa and IL-6 production in the serum of recipient mice. Strikingly, in a murine model of adoptive transfer of autoimmune diabetes, miR-29b reduces the cytolytic activity of transferred effector CD8+ T-cells, insulitis and disease incidence in a single standalone intervention. Endogenous miR-29b, spontaneously released from beta-cells within exosomes, stimulates TNFa secretion from spleen cells isolated from diabetes-prone NOD mice in vitro. Hence, microRNA sequences modulate innate and ongoing adaptive immune responses raising the question of their potential role in the breakdown of tolerance and opening up new applications for microRNA-based immune therapy.

## Introduction

MicroRNAs (miRNAs) are abundant, highly conserved, 18–24 nucleotides-long, non-coding RNAs. MiRNAs are known to post-transcriptionally regulate up to hundreds of genes by more or less perfect base pairing with target messenger RNAs leading to repression of translation, a process termed RNA interference (RNAi). Through RNAi, miRNAs control all fundamental biological processes like differentiation, proliferation, apoptosis, morphogenesis, inflammation, immune- and metabolic pathways [1]. MiRNAs also participate in intercellular communication after release into the extracellular space inside membrane vesicles or lipo-protein complexes that protect them against degradation. Exosomes are 40–100 nm sized membrane vesicles that transport functional mRNA, miRNAs and proteins from their cell of origin towards recipient cells [2], [3]. Evidence emerges that extracellular miRNA sequences can also bind to RNA-sensing receptors of the toll-like receptor (TLR) family, independently of RNAi: in a mouse model of Alzheimer’s disease, the endosomal receptor TLR-7 was identified as a key element for mir-let-7b mediated immune-stimulation exacerbating neurodegeneration [4]. Similarly, tumour-secreted miR-21 and miR-29a trigger prometastatic and inflammatory responses in macrophages through human TLR-8 or mouse TLR-7 signalling [5]. On the contrary, TLR-1 rather than TLR-7/8 seems to be involved in miRNA immune activation of natural killer (NK) cells, suggesting cell-specific pathways [6]. Whether miRNA-mediated immune-stimulation may fuel autoimmune responses has not been addressed yet.

Type 1 diabetes (T1D) is a chronic autoimmune disorder that results from the specific destruction of insulin-producing pancreatic beta cells by autoreactive T-lymphocytes, especially CD8^+^ T-lymphocytes [7]. The mechanisms underlying the initiation and progression of the disease are poorly understood, but seem to involve the breakdown of multiple tolerance networks. To date, it is a well established fact that susceptible individuals have a complex multigenic predisposition and that environmental triggers i.e. enteroviral infections may lead to enhanced beta-cell apoptosis, dendritic cell (DC) activation and subsequent T-cell priming [8]. Immune complexes containing self nucleic acids, DNA or RNA, contribute to autoimmunity in systemic lupus erythematosus, psoriasis, polyarthritis, and diabetes [9]–[11]. Aberrant miRNA expression patterns have been associated with disease progression in T1D patients [12], [13]. Whether miRNA missexpression is merely a consequence of T1D or whether miRNAs participate in disease development remains to be investigated.

Here we report that some pancreatic beta-cell miRNA analogues are immune-active molecules, able to drive pro-inflammatory (TNFa, IFNg, IL-6, IL-12) as well as suppressive (IL-10) cytokine secretion from DC *in vitro* and *in vivo*, in a sequence-dependent manner. Further investigation in the murine RAW264.7 macrophage cell line supports that, for the miR-29b, immune modulation is mediated by TLR-7, independently of RNAi activity. *In vivo*, the systemic delivery of miR-29b dampens antigen-specific T-cell responses and reduces disease incidence in a murine model of adoptive transfer of autoimmune diabetes. *In vitro* generated beta-cell exosomes enclose specific miRNA sequences including miR-29b. These beta-cell exosomes stimulate TNFa, IL-6, and IL-10 cytokine secretion from splenocytes isolated from diabetes-prone NOD mice *in vitro*. TNFa secretion is impaired in the presence of miR-29 inhibitors. Our results demonstrate that, in addition to their potent effect as regulators of gene expression, some beta cell miRNA sequences may act as modulators of innate and adaptive immune responses opening new possibilities for miRNA-based immune intervention in autoimmune diseases such as T1D.

## Materials and Methods

### Ethical statement

All cares and experiments with animals (Mice) were carried out in strict accordance with relevant French guidelines (Décret 2001-464, 29 mai 2001 and Décret 2013-118, 1er février 2013). Animals were housed in the ONIRIS’ Rodent Facility (Agreement Number: 44 266) in a specific pathogen-free environment (MICE™, Charles River Laboratories, Wilmington, MA, USA) with sterilized tap water and food. All animal experiments were carried out under the responsibility of staff accredited by the *Direction Départementale de la Protection des Populations/Expérimentation animale (J.M.B. – Agreement Number: 44 84)*, and procedures on animals were approved by the *Pays de la Loire* regional Committee on the Ethics of Animal Experiments (Permit Number: CEEA.2012.251). All efforts were made to minimize suffering.

### Mice and diabetes

BALB/c mice were obtained from JanvierLabs (Le Genest Saint Isle, France). Female mice from all strains were used between 8–12 weeks of age. Thy1.2 (CD90.2) H-2K^d^ Ins-HA and CL4-TCR transgenic mice, kindly provided by Pr Roland LIBLAU (INSERM U1043, Toulouse University Hospital, France), were used for diabetes transfer experiments. Ins-HA transgenic mice express the hemagglutinin A (HA) protein of the influenza virus “A PR8 34”, under the control of the rat insulin promoter specifically in pancreatic beta cells. In CL4-TCR mice, 95% of peripheral CD8^+^ T-cells express a transgenic CD8^+^ TCR specific for the H-2K^d^-restricted peptide HA_512–520_ (IYSTVASSL) [14]. CL4-TCR and Thy1.1 (CD90.1) BALB/c mice (CDTA, Orleans, France) were mated to obtain CL4-TCR^+^Thy1.1^+^ mice. Autoimmune diabetes was transferred to Ins-HA recipient mice through the intravenous injection of HA-specific CTLs from CL4-TCR mice. One BALB/c and one CL4-TCR donor mouse was used in each transfer experiment. For *in vivo* tracking, transferred cells were generated from CL4-TCR^+^Thy1.1^+^ mice. Diabetes was monitored using Clinistix strips for urinalysis (Bayer HealthCare, Puteaux, France) and a Glucotrend/Accu-Chek glucometer (Roche Diagnostics, Mannheim, Germany). Mice were considered diabetic when blood glucose levels were >11 mM on two consecutive days. NOD/ShiLtJ mice were purchased from Charles River Laboratories (L’Arbresles, France). Female mice were used before diabetes onset at six to ten weeks of age.

### Cells

The RAW 264.7 murine macrophage cell line (ATCC nb TIB-71) was cultured in RPMI 1640 medium (Life Technologies, Saint Aubin, France) supplemented with 10% fetal calf serum (FCS) and 2 mM L-glutamine. RAW cells were plated at 33×10^3^ cells/well, in 96-well plates, four hours before TLR-ligand or miRNA treatment (adapted from [15]). Supernatants were collected eighteen hours later and analysed for TNFa secretion by ELISA. BmDCs were generated from BALB/c bone-marrow as described by Lutz *et al*. [16] with slight modifications. Briefly, bone-marrow precursor cells were extracted from femur and tibia bones and were cultured for six days in complete RPMI 1640 medium (10% FCS, 2 mM L-glutamine, 100 IU/ml penicillin, 100 mg/ml streptomycin, 1 mM sodium pyruvate, non essential amino acids, and 20 µM beta-mercapto-ethanol), supplemented with 20 ng/ml of GM-CSF (R&D Systems, Minneapolis, MN, USA). CD11c^+^ DCs were purified by positive selection with magnetic mouse CD11c microbeads (Miltenyi Biotec, Bergisch Gladbach, Germany). Cell purity was routinely assessed as >95% by flow cytometry. For cytokine secretion assays, CD11c^+^ bmDCs were plated in 24-well plates at 2×10^5^ cells/well.

The MIN6 cell line, kindly provided by Prof. Jun-ichi Miyazaki (University Medical School, Osaka, Japan), was cultured in DMEM high glucose medium (Life Technologies) supplemented with 10% FCS [17].

Splenocytes were isolated from NOD/ShiLTJ mice by gentle mechanical disruption of the spleen, passing through a 100 µm sieve, followed by lysis of the red blood cells. For cytokine secretion assays, splenocytes were plated at 8×10^5^ cells per well in 96 flat-bottom wells in 200 µl of medium (RPMI 1640, 10% FCS, 2 mM L-glutamine, 100 IU/ml penicillin, 100 mg/ml streptomycin) pre-cleared from FCS serum exosomes using 100,000×g overnight pre-centrifugation [18].

Activated HA-specific CD8^+^ T-cells were obtained as previously described [19], with some modifications. Briefly, CD8^+^ lymphocytes were purified from spleens of CL4-TCR mice using CD8 positive magnetic cell sorting (Miltenyi). Cell purity was routinely >95% as assessed by flow cytometry. 1×10^6^ CD8^+^ T-cells were stimulated with 5×10^6^ mitomycin C treated BALB/c splenocytes (Sigma-Aldrich, St Louis, MO, USA), with 5 µM HA_512–520_ peptide (ProImmune, Oxford, UK), 5 U/ml rhIL-2 (Roche Applied Science, Penzberg, Germany) and 20 ng/ml rmIL-12 (R&D Systems), in 2 ml complete DMEM medium in 24-well plates. Cells were collected on day 4 for intravenous injection in recipient Ins-HA mice or were seeded onto 24-well plates (3 or 15×10^5^ cells/well) for *in vitro* transfection experiments.

### MiRNA analogues and transfection experiments

We used synthetic ds-miRNA analogues (F/R), composed of the mature miRNA guide strand sequence (F) and its complementary reverse strand (R). 3′-overhangs were eliminated in order to prevent an interfering effect, as 3′-overhangs appear to support this function [20]. MiRNA analogues, as well as 2′-O-Methyl (2′-O-Me) -modified miRNA sequences were synthesized by Eurogentec (Seraing, Belgium) and tested for endotoxins (<5 EU/mg). Ds-miRNAs were obtained by annealing ss-miRNA sequences according to the supplier’s instructions.

For immune monitoring *in vitro*, miRNAs and controls were complexed to DOTAP Liposomal Transfection Reagent (Roche Applied Science) at a 0,16 ARN:DOTAP (µg:µl) ratio and used at a final concentration of 150 nM for DC transfection or at a 0,31 ARN:DOTAP (µg/µl) ratio at indicated concentrations in RAW264.7 and splenocyte experiments. For *in vivo* use, 10 µg per mouse of miRNAs in 100 µl Hepes-buffered saline (HBS) were embedded in 100 µl DOTAP before injection in the lateral tail vein. SiRNA9.2 (5′-AGCUUAACCUGUCCUUCAA-3′, 5′-UUGAAGGACAGGUUAAGCU-3′) and siRNA9.1 (5′-UGGACGGCAACUGUUAUUA-3′, 5′-UAAUAACAGUUGCCGUCCA-3′) sequences described earlier [21] (Eurogentec) served as positive and negative controls, respectively. For *in vitro* ARN interference assays, 1×10^5^ RAW264.7 macrophages were plated per well onto 24-well plates the day before transfection. SiMcl1 (5′-UAGCACCAUGGUUAAGACUCUdTdT-3′) and siRNA negative control from Eurogentec were transfected at a final concentration of 2.7 µM using Viromer blue (Lipocalyx, Halle, Germany) and the supplier’s sense protocol.

For miR-29 knockdown, locked nucleic acid (LNA) miRNA-29 family inhibitor and LNA negative control were purchased from Exiqon (Exiqon, Vedbaek, Denmark). Exosomes were transfected over-night with Exofection (Gentaur, Paris, France) and harvested using the PureExo Isolation Kit (Gentaur) following the supplier’s instructions.

### Isolation of Exosomes

Exosomes were collected from supernatants from MIN6 cells (15×10^4^ cells/cm^2^) cultured in medium pre-cleared from serum exosomes using differential centrifugation and one PBS wash step [18]. The final pellet was dissolved in 1 µl PBS per ml of initial culture supernatant.

### Antibodies and reagents

Phenotypic analysis of mouse DCs, NK cells, CD8^+^ T-cells and CD4^+^ T-cells was performed by flow cytometry (FACS Aria, BD Biosciences, Le Pont de Claix, France) using DiVa (BD Biosciences) and FlowJo softwares (Tree Star Inc., Ashland, OR, USA). mAb used were: CD11c (HL3), CD45R/B220 (RA3-6B2), CD11b (M1/70), CD40 (3/23), CD86 (GL1), H-2Kd (SF1-1.1), CD49b/Pan-NK (DX5), CD8 (53–6.7), CD4 (RM4-4), CD69 (H1.2F3), CD90.1/Thy-1.1 (HIS51) (all from BD Biosciences), and CD3 (145-2C11) (Beckman-Coulter, Fullerton, CA, USA), as well as adequate isotypic controls. Imiquimod (TLR-7 ligand, 10 µg/ml, Invivogen, San Diego, CA, USA), R848 (TLR-7/8 ligand, 0.1 µg/ml, Invivogen) and lipopolysaccharide (LPS, TLR-4 ligand, 1 µg/ml, Sigma-Aldrich) served as TLR agonists. The IRS661 5′-TGCTTGCAAGCTTGCAAGCA-3′ with phosphorothioate backbone modifications (Eurogentec) and chloroquine (Sigma-Aldrich) were used at 5 µM and 10 µM working concentrations, respectively. IFNa was quantified using the Verikine Mouse Interferon-Alpha ELISA kit (R&D Systems). Other cytokines, namely IL-12p70, IL-10, and TNFa were quantified using mouse DuoSet ELISA kits (R&D Systems). Analysis of the cytokines IL-6, TNFa, IL-1b, IL-10 and IL-12 in mice sera was performed using BD Cytometric Bead Array Flex Sets (BD Biosciences), according to the supplier’s protocol. Samples were acquired on a FACS Aria and analysed using the FCAP Array Software (BD Biosciences).

### Confocal microscopy

RAW264.7 cells were plated on Lab-Tek II chambered coverglasses at a density of 7×10^5^ cells/cm^2^ on the day before transfection. Transfection was carried out using 500 nM of miRNA analogues. Cells were fixed with 4% paraformaldehyde, permeabilized with 0,2% Tween20, and incubated with 5% goat blocking serum in PBS and incubated with 1 µg/ml primary rabbit anti- mouse EEA-1 antibodies (Abcam, Paris, France) for 1 hour at 37°C followed by incubation with 8 µg/ml secondary goat anti-rabbit ALEXA-555-conjugated antibody for 45 min. at room temperature. Alternatively, living cells were stained with 75 nM Lysotracker (Fischer Scientific). Cells were counterstained with 5 µM DRAQ5 (eBioscience, Paris, France) and overlayed with Mowiol medium (Biovalley, Conches, France). Confocal imaging was performed on an inverted Nikon TE-2000 laser scanning confocal microscope (Nikon, Champigny, France).

### *In vivo* cytotoxicity assay

Cytolytic activity of activated HA-specific CD8^+^ T-cells was assessed *in vivo* [22]. Briefly, splenocytes were obtained from BALB/c mice. The target population was pulsed with 5 µM HA_512–520_ peptide and labelled with 15 µM CFSE (Life Technologies) against the unpulsed control population labelled with 1.5 µM CFSE. 48 h after HA-specific CD8^+^ T-cell injection, 5×10^6^ cells of the pulsed and unpulsed populations were injected intravenously in a 1:1 ratio in recipient Ins-HA mice. Splenocytes of recipient Ins-HA mice were harvested sixteen hours later, and cytolytic activity was assessed by flow cytometry. Specific lysis (%) = 100×[(CFSE_low_−CFSE_high_)/CFSE_low_].

### Assessment of insulitis

Sections of 7 µm of frozen pancreata were stained with hematoxylin and eosin and the degree of insulitis was rated independently by two investigators in a blinded fashion on a total of >100 islets.

### Statistical analysis

Statistical analyses were performed using Prism (GraphPad Software, Inc.) and statistical tests indicated in figure legends.

## Results

### MiRNAs stimulate cytokine secretion by mouse antigen-presenting cells (APCs) in a sequence-dependent manner, *in vitro* and *in vivo*

It has been recently demonstrated that certain miRNA sequences can induce inflammatory responses *in vitro* and *in vivo* following sensing by TLRs [4]–[6]. To test the hypothesis that beta-cell miRNA sequences can modulate immune responses, ten miRNA sequences were chosen for their selective abundance in murine pancreatic beta cells [23], [24]. The mature miRNAs miR-16 and miR-29b are also present in immune cells (DCs and T- and B-lymphocytes) and in other cell types (alpha pancreatic cells, neuroblasts, kidney duct cells, testis/ovary…). Let-7c and miR-30d are expressed in brain, neural, kidney, and reproductive organ cells, but only weakly in immune cells. MiR-375, -127, -7a, -210, -129-5p and -376a are mostly present in pancreatic endocrine cells.

**Figure 1:**
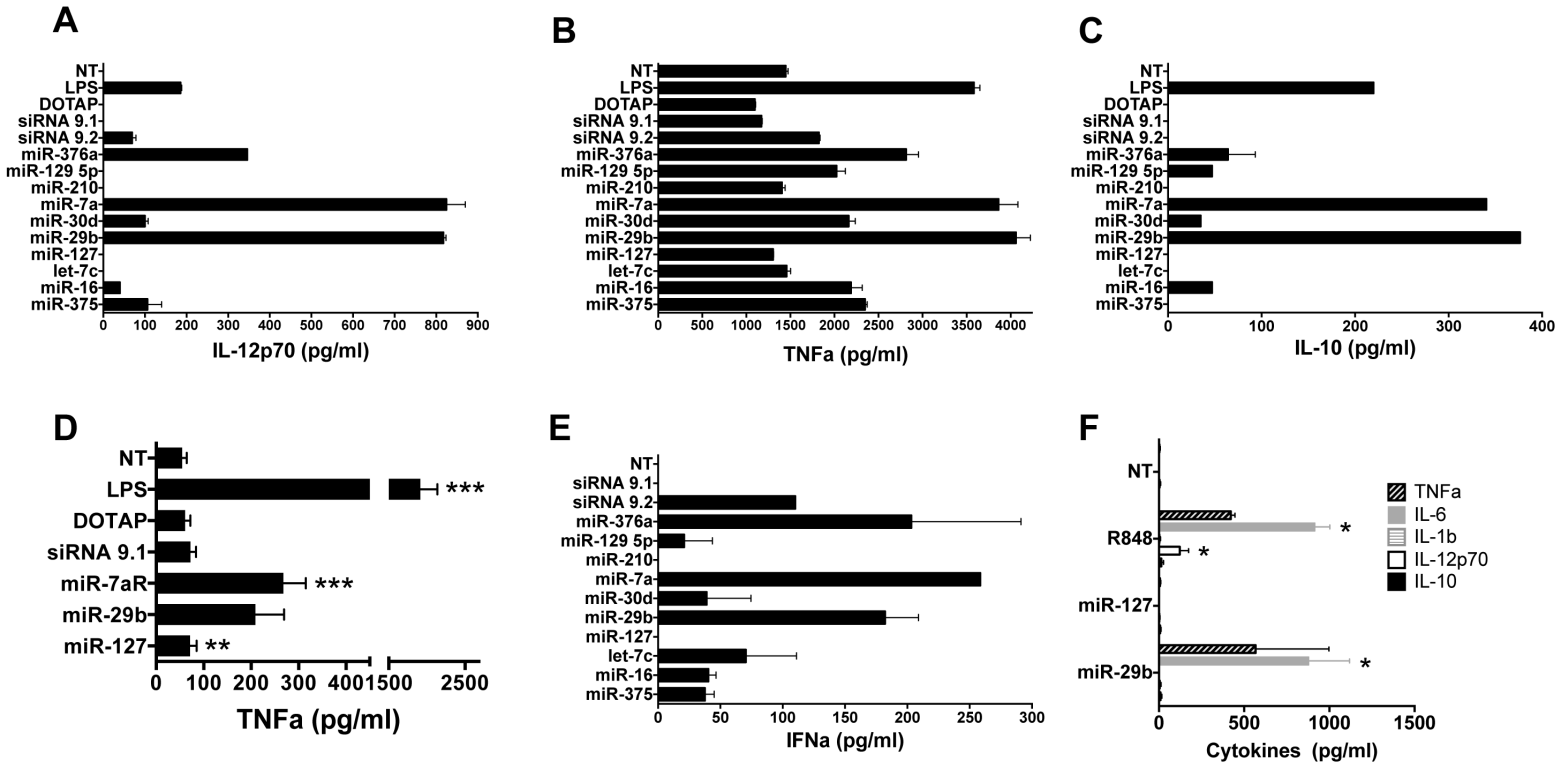
Cytokine secretion induced by miRNAs *in vitro* and *in vivo*. Purified mouse CD11c^+^ bmDCs or RAW264.7 mouse macrophages were stimulated *in vitro* with miRNAs complexed to DOTAP at a working concentration of 150 nM (bmDCs) or 750 nM (macrophages, positive controls (siRNA9.2 or LPS), negative controls (siRNA9.1 or DOTAP alone) or were left untreated (NT). IL-12 (A), TNFa (B) and IL-10 (C) cytokine levels were assessed by ELISA in bmDC supernatants eighteen hours after stimulation. Results are presented as mean cytokine concentration of duplicates (pg/ml) ± SEM. Data from one representative experiment out of two is shown. (D) TNFa secreted by RAW264.7 macrophages was quantified in supernatants after eighteen hours of stimulation. Results compiled from four independent experiments are shown and analysed using a Kruskal-Wallis test (***P<0.001 and **P<0.01). (E) Serum IFNa in BALB/c mice was quantified by ELISA seven hours following intravenous injection of miRNAs complexed to DOTAP or controls. Results are presented as mean concentration of duplicates (pg/ml) ± SEM, and are confirmed in a second independent experiment (n = 4 total mice). (F) Serum IL-6, TNFa and IL-12 in BALB/c mice was quantified two hours following intravenous injection of miR-29b, the positive control R848, or the immune-silent miR-127 using a BD Cytometric Bead Array. Results are presented as mean concentration ± SEM (pg/ml) from two experiments (n = 4 total mice). IL-6: *P*<0.05 for miR-29b vs miR-127 and miR-127 vs R848; IL-12: *P*<0.05 for miR-127 vs R848 (Kruskal-Wallis).

MiRNA analogues were first tested for their ability to induce innate cytokine secretion in mouse bone-marrow-derived dendritic cells (bmDCs) *in vitro*. Three miRNA sequences, namely miR-29b, miR-7a, and miR-376a induced IL-12 secretion (Fig. 1A) and enhanced basal TNFa secretion (Fig. 1B), exceeding levels obtained for LPS and siRNA9.2 [21] positive controls. Interestingly, secretion of the anti-inflammatory cytokine IL-10 was also observed for miR-29b and miR-7a (Fig. 1C). Notably, miR-127 and miR-210 were consistently immune-silent in all cytokine assays, whereas other miRNA sequences induced weak to moderate responses (Fig. 1A–C). Sequence-specific stimulation of TNFa secretion for mir-7a and 29b was confirmed in RAW264.7 mouse macrophages *in vitro* (Fig. 1D). Again, miR-29b (p<0.01) and miR-7a (p<0.001) significantly increased TNFa secretion compared to untreated cells in contrast to miR-127 and negative controls. MiRNA-mediated immune stimulation is dose-dependent with highest TNFa secretion observed for miR-29b at 500 nM (p<0.05) and 750 nM (p<0.01), whereas miR-127 induced no significant dose-response changes compared to untreated cells (S1A in File S1). These working concentrations are in line with *in vitro* immune monitoring assays developed earlier [15].

*In vivo*, immune stimulation was assessed through serum IFNa content, seven hours after intravenous delivery to BALB/c mice (Fig. 1E). Again, miR-29b, miR-7a and miR-376a stimulated IFNa production in sera of treated mice, in contrast to miR-127 and miR-210. The expression of miR-29b in islet cells increases with age in the spontaneous Non-Obese Diabetic (NOD) mouse model of autoimmune diabetes and an over-expression of miR-29b is observed in mouse and human islet cells following exposure to pro-inflammatory cytokines [25]. Hence, the immunogenic miR-29b was selected in pursuit of these results for more in depth analysis of the underlying immune modulatory mechanisms. The immune-silent miR-127 served as negative control. The cytokine profile in serum was completed by testing the effect of the miR-29b on IL-1b, IL-6, IL-10, IL-12 and TNFa secretion, two or seven hours following its injection in BALB/c mice. As shown in Fig. 1F and Table 1, miR-29b but not miR-127 greatly albeit transiently stimulated IL-6 and TNFa secretion in sera two hours after injection. In contrast to the control TLR-7- agonist R848, no IL-12 secretion was observed following miR-29b delivery. For all situations, no IL-1b or IL-10 was observed at any time of analysis. Preliminary data obtained using a pDC-depleting antibody before miR-29b administration led to a >80% decrease in IFNa concentration (from 343 pg/ml to 57 pg/ml) (S2 in File S1), suggesting a direct or indirect effect of miR-29b on pDC-mediated production of IFNa *in vivo*.

**Table 1:** Cytokine profile in BALB/c mice serum after intravenous miRNA delivery.

		miR-29b	miR-127	R848	HBS
2 h	IL-6	878.1±480.6	nd	914.5±176.1	nd
	TNFa	566.6±430.9	5.7±4.4	421.3±24.6	2.6±4.3
	IL-12p70	4.2±8.1	2.0±3.9	121.6±52.4	3.1±6.2
7 h	IL-6	88.9±103.4	nd	nd	nd
	TNFa	29.6±31.9	4.3±5.2	15.9±7.3	7.5±8.6
	IL-12p70	35.9±32.0	nd	26.5±21.0	3.1±6.2

Cytokine content in serum from BALB/c mice was analysed by a BD Cytometric Bead Array two and seven hours following intravenous injection of miR29b, the immune-silent miR-127 or positive (R848) or negative (HBS) controls. Results are presented as mean concentration (pg/ml) ± SEM from two experiments (n = 4 total mice); nd: not detected.

Taken together, our results show that beta-cell miRNA analogues exert a potent stimulatory effect on cytokine production by APCs, in a sequence-dependent manner.

### Mouse macrophage stimulation by miR-29b involves endosomal TLR-7, independently of RNA interference

**Figure 2:**
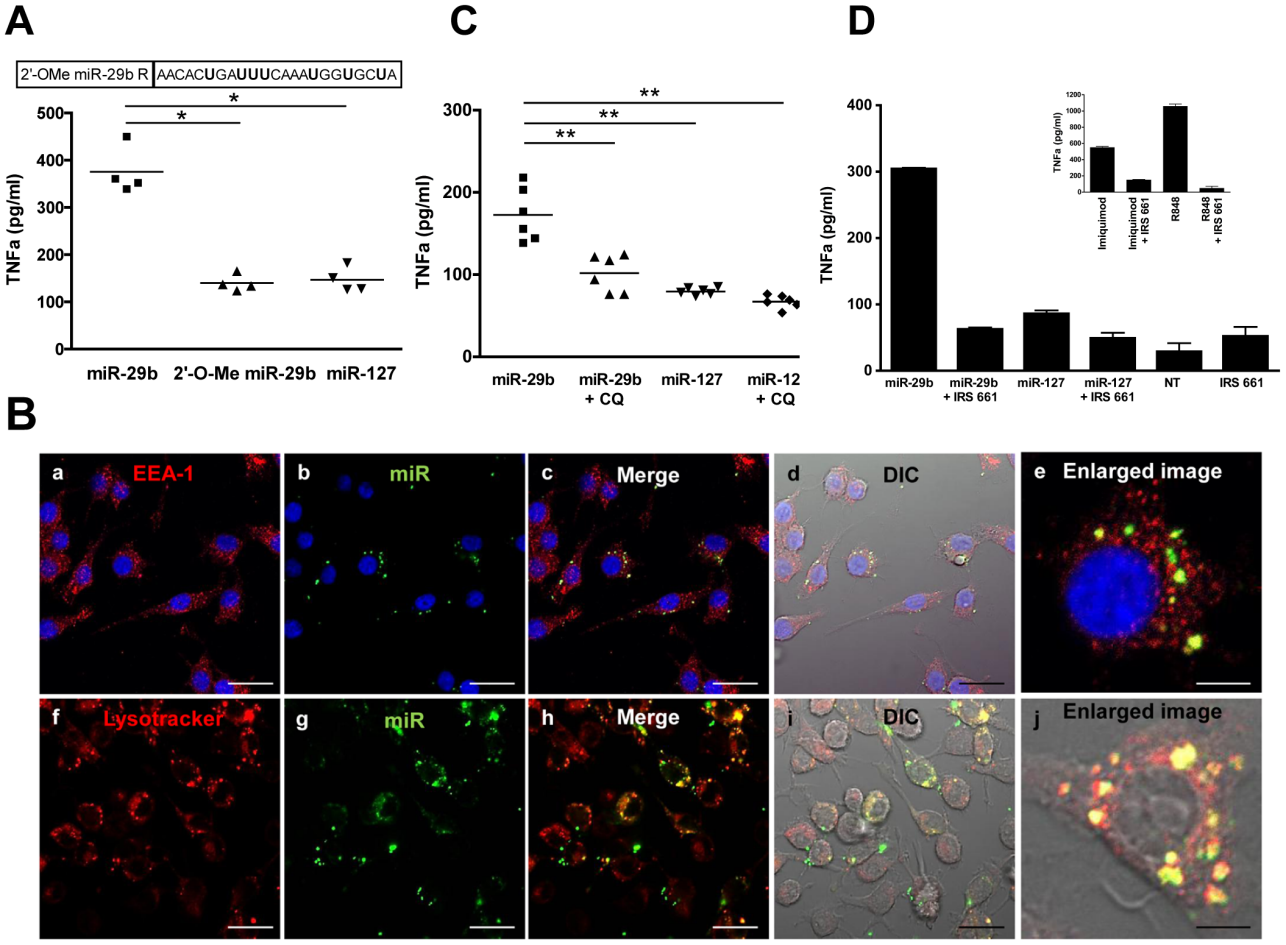
Stimulation of the TLR-7 pathway by miR-29b in murine RAW264.7 macrophages. (A) 2′-O-methyl modifications were introduced in all uracil residues of the miR-29b reverse strand as indicated. RAW264.7 cells were plated four hours before stimulation with DOTAP-embedded miR-29b, 2′-O-Me-modified miR-29b, or the control miR-127 (750 nM working concentration). TNFa was quantified in supernatants eighteen hours later. 2′-O-Me modifications were introduced in the miR-29b reverse strand before annealing to the unmodified guide strand. Results are represented as individual values of cytokine concentrations (pg/ml). Data from one representative experiment out of three is shown. *P<0.05 (Mann-Whitney). (B) Intracellular distribution of Alexa-488-labelled miR-29b 1hour after transfection of RAW264.7 cells was observed with a confocal fluorescence microscope. Top row: IF EEA-1 on fixed cells Bottom row: lysotracker in living cells. Scale bar = 20 µm for fluorescence images and overlays with differential interference contrast (DIC) (a–d, f–i) except for enlarged single cell images scale bar = 5 µm (e, j). (C) Chloroquine (CQ) was added to the plated RAW264.7 cells, at a final concentration of 10 nM, 30 minutes before stimulation with miR-29b or the miR-127 control (750 nM). Supernatants were harvested eighteen hours later for TNFa quantification. Results are represented as individual values of cytokine concentrations (pg/ml) compiled from two independent experiments. **P<0.01 (Mann-Whitney) (D) RAW264.7 cells were stimulated with miR-29b, miR-127 (750 nM), the positive controls TLR-7-ligand imiquimod and R848, or were left untreated (NT), and were cultured eighteen hours with or without the TLR-7 antagonist IRS661. TNFa was quantified in supernatants. Results are presented as mean cytokine concentration of replicates (pg/ml) ± SEM. Data from one representative experiment out of three is shown.

To discriminate between RNAi-mediated immune effects and direct immune stimulation, 2′-O-Me modifications were introduced in each uridine base on the reverse strand of the miR-29b sequence (Fig. 2A). These modifications have been described to impede direct TLR activation, without alteration of RNA silencing activity [26]. As shown in S3 in File S1), 2′-O-Me modifications do not impact the RNAi activity of the miR-29b analogue. Yet, 2′-O-Me modifications in the miR29b sequence led to a significant drop in TNFa secretion by RAW264.7 cells (p<0.05), close to control levels, indicating a RNAi-independent process (Fig. 2A).

As innate immune receptors differ in their aptitude to recognise double-stranded or single-stranded nucleic acids, miR-29b duplex or single-stranded sequences were compared for their respective effects on TNFa secretion by RAW264.7 cells (S1B in File S1). In our hands, the forward and reverse miR-29b strands induced similar TNFa secretion than their double-stranded counterpart. This result may be readily explained by the binding of a single-stranded intermediate, although we cannot definitively rule out a distinct pathway involving a double-stranded ligand.

Whether the exogenous miR-29b enters the endosomal pathway was studied using confocal microscopy in RAW264.7 cells. One hour after transfection, an ALEXA-488-labeled miR-29b co-localizes with the endosomal markers Early Endosomal Antigen 1 protein (EEA-1) and lysotracker (Fig. 2B). Chloroquine has been described to prevent endosomal TLR activation by nucleic acids either by inhibiting the acidification of endosomes associated with TLR7/8 activation or by modifying the three-dimensional TLR conformation [27]. Chloroquine added to RAW264.7 cells prior to miRNA transfection clearly inhibited TNFa secretion (p<0.01, Fig. 2C). As chloroquine does not affect cell viability at the working concentration used (data not shown), this result points to the involvement of the endosomal pathway in the miR-29b’s immune activity.

To determine whether miR-29b stimulation relies on TLR-7, we used the immune-regulatory sequence IRS661, a competitive inhibitor of TLR-7 binding [28]. In RAW264.7 cells, IRS661 reduced miR-29b-induced TNFa secretion by 80% (Figure 2D). In one representative experiment out of three, TNFa secretion decreased from 304.2±2.3 pg/ml to 62.6±3.6 pg/ml after IRS661 inhibition. IRS661 also specifically impaired imiquimod and R848 stimulation, two reference TLR-7 agonists [29], [30].

### MiR-29b inhibits *in vivo* adoptive transfer of autoimmune diabetes by CD8^+^ T-cells

**Figure 3:**
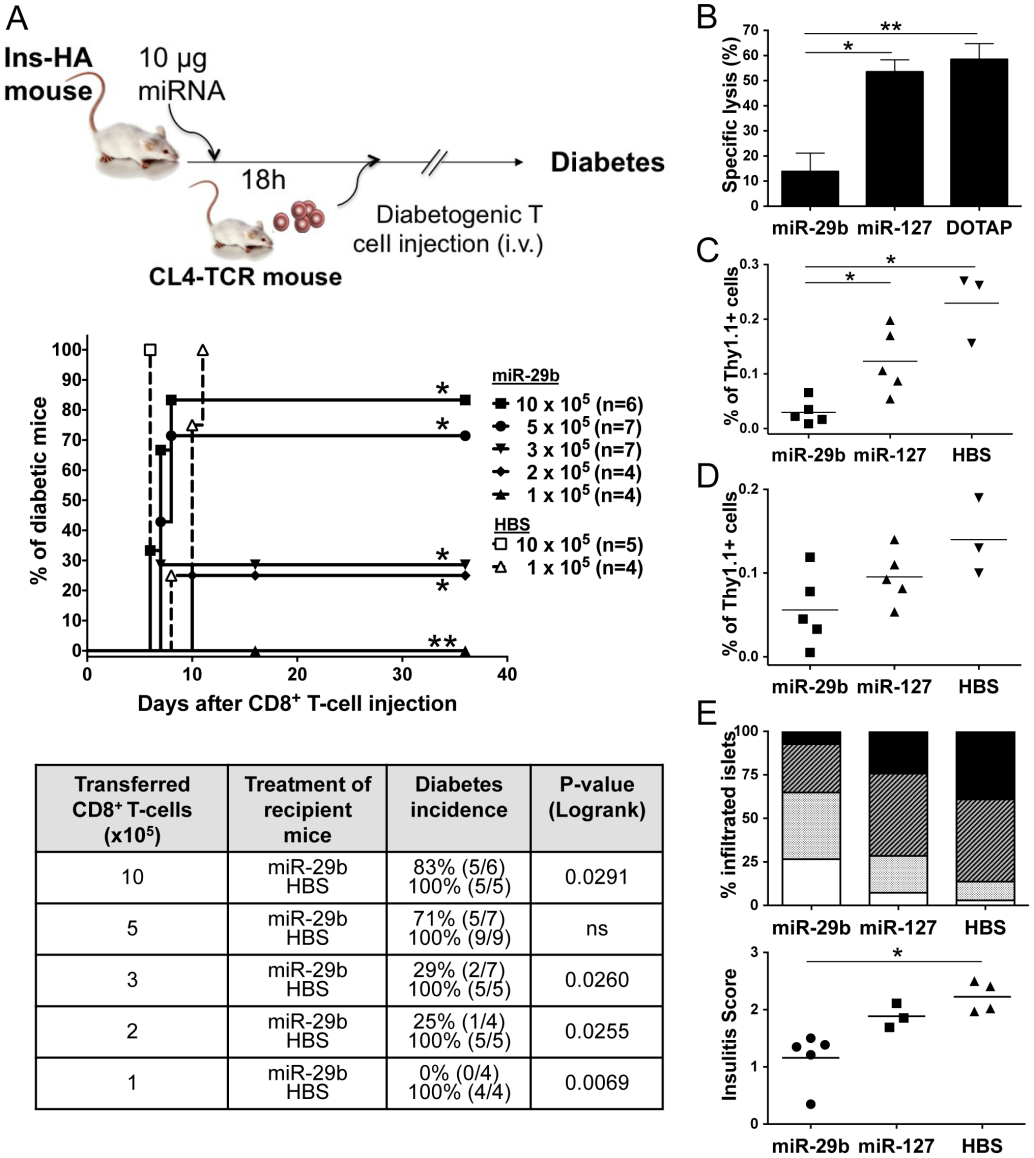
Systemic delivery of miR-29b protects against adoptive transfer of T1D *in vivo*. Ins-HA mice were treated intravenously with miR-29b, miR-127, HBS buffer or DOTAP alone, eighteen hours before receiving HA-specific CTLs from CL4-TCR mice. (A) Recipients were monitored for diabetes development for at least one month. The survival curves and table summarize the results of five independent experiments after transfer of 1 to 10×10^5^ cells, with miR-29b -injected mice as filled symbols, and HBS-injected mice as empty symbols. The table indicates, for each group, the percentage of final cumulative diabetes incidence and the number of diabetic mice among all mice in the group in brackets. A logrank test was performed for statistical significance of differences between Kaplan-Meier incidence curves. (B) Eighteen hours after miRNA injection, Ins-HA recipient mice received 5×10^5^ activated HA-specific CTLs, followed 48 h later by the intravenous administration of HA-pulsed «CFSE_high_ » and non-pulsed «CFSE_low_ » target cells mixed at a 1∶1 ratio. Splenocytes from recipient Ins-HA mice were analysed by flow cytometry, sixteen hours after target cell injection. The bar chart shows the compiled results of three independent experiments (n = 4–5 mice/group) as mean specific lysis ± SEM. *P<0.05, **P<0.01 (Mann-Whitney). (C–E) Eighteen hours after miRNA injection, Ins-HA were transferred with 8×10^5^ activated HA-specific Thy1.1^+^ CTLs from CL4-TCR^+^Thy1.1^+^ mice. Four days later, spleens (C) and PLNs (D) were harvested from Ins-HA recipient mice and analysed by flow cytometry. Compiled results of two independent experiments are presented as the percentage of Thy1.1^+^ cells in individual mice gated on the CD3^+^ CD8^+^ T-cell population (n = 3–5 mice), and were confirmed in a third experiment. *P<0.05 (Mann-Whitney). (E) Histological analysis of insulitis of pancreata: 0 = islet free of mononuclear cell infiltration (unfilled bars); 1 = peri-insular infiltration involving <10% of the islet area (punctuated bars); 2 = infiltration involving between 10% and 50% of the islet area (hatched bars); 3 = infiltration involving >50% of the islet area (black bars). The stacked vertical bar graph indicates the percentage of islets in each category described above. Results are presented as the mean percentage of n = 5 mice for miR-29b, n = 3 for miR-127, and n = 4 mice in the HBS group from three independent experiments. For each pancreas, an average insulitis score was calculated by adding up the score of each islet and dividing it by the total number of islets counted. Results show the individual insulitis scores for each group of recipient mice. *P<0.05 (Kruskal-Wallis).

With the aim to investigate the effect of the miR-29b analogue on T-cell effector functions *in vivo*, we used the Ins-HA transgenic mouse model of autoimmune diabetes [14]. Briefly, 1 to 10×10^5^ activated HA–specific CD8^+^ T-cells from CL4-TCR mice were transferred to Ins-HA recipient mice previously injected with miR-29b, miR-127, or HBS negative control (Fig. 3A). Monitoring of diabetes showed consistently a 100% disease incidence for mice injected with HBS alone, at any given dose of T-cells injected. Similarly, mice injected with miR-127 after transfer of 3×10^5^ or 5×10^5^ CD8^+^ T-cells all developed diabetes (data not shown). In contrast, only 83% of miR-29b-treated mice became diabetic after the injection of 1×10^6^ T-cells (p<0.03), and no diabetes was observed after transfer of 1×10^5^ T-cells (p<0.01). In conclusion, miR-29b was able to decrease the antigen-specific pathogenic activity of effector CD8^+^ T-cells and to confer protection against diabetes outbreak.

### MiR-29b reduces the cytolytic activity and persistence of effector CD8^+^ T-cells *in vivo*

How miR-29b reduced disease incidence was investigated by *in vivo* cytotoxicity experiments (Fig. 3B). Briefly, Ins-HA mice were injected with activated HA-specific CD8^+^ T-cells followed by the injection of HA-pulsed spleen target cells. In control mice, miR-127 or DOTAP treatment resulted in 53.5±4.8% or 58.5±6.2% target cell lysis, respectively. In contrast, a specific lysis of only 13.8±7.3% occurred in miR-29b mice (p<0.05 versus miR-127 and p<0.01 versus DOTAP). These data suggest that miR-29b alleviates diabetes through decreased cytolytic activity of the injected CTLs. A possible explanation for this decrease in miR-29b-injected mice may be a deletion of effector CD8^+^ T-cells. To address this question, HA-specific Thy1.1^+^ CD8^+^ T-cells were quantified in spleens (Fig. 3C) and pancreatic lymph nodes (PLNs) (Fig. 3D) four days after transfer to recipient Thy1.2 Ins-HA mice. Cell recovery sufficient for donor cell quantification requires injection of 8×10^5^ Thy1.1^+^ CD8^+^ T-cells. Mice were euthanized before diabetes onset and the percentage of Thy1.1^+^ cells in spleens and PLNs was assessed by flow cytometry in the CD3^+^CD8^+^ T-cell population. A significant decline in the number of Thy1.1^+^ cells was observed in the spleen of miR-29b-injected mice, compared to miR-127 and HBS controls (p<0.05). This decrease was not due to a difference in the homing to PLNs, because only a slight and not significant difference in the number of Thy1.1^+^ cells was observed in PLNs. Finally, pancreatic islet infiltration four days after transfer is less invasive in miR-29b treated mice as shown by histological analysis (Fig. 3E). In conclusion, these results argue in favour of a decrease in the absolute number of Thy1.1^+^ cells after transfer, conferring protection against insulitis and overt diabetes, rather than an absence of T-cell migration to the pancreas.

### MiR-29b activates immune cells *in vivo*

**Figure 4:**
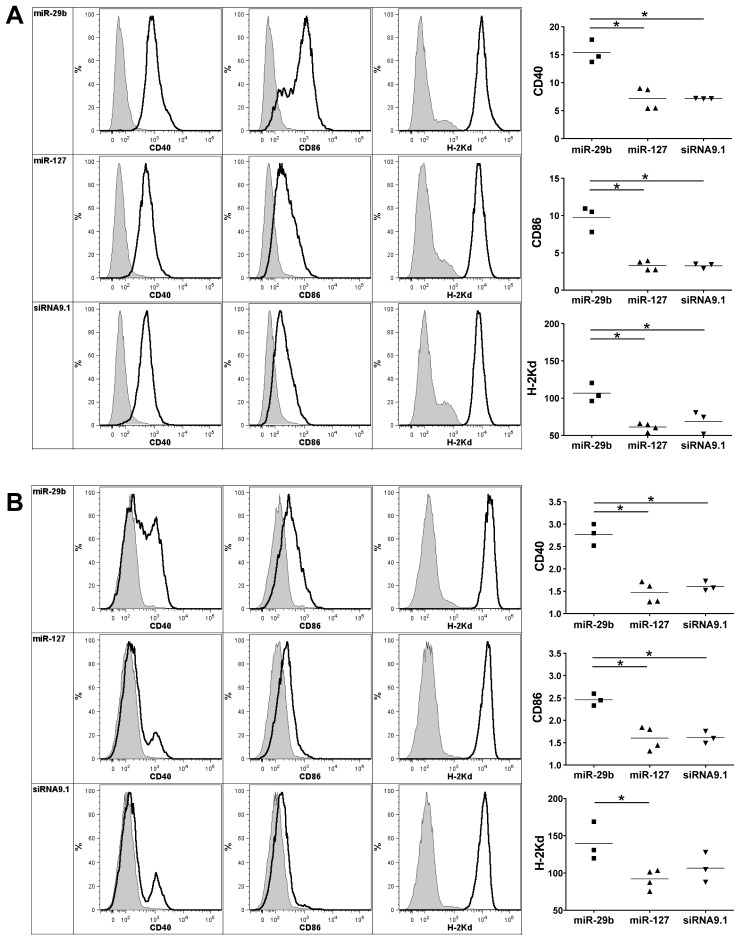
Splenic mDC and pDC activation by miR-29b *in vivo*. BALB/c mice were injected intravenously with miR-29b, miR-127, or siRNA9.1. Spleens were harvested eighteen hours after injection and CD40, CD86, and H-2Kd expression was evaluated by flow cytometry, on CD11c^+^CD11b^+^B220^−^ mDC (A) or CD11c^low^CD11b^−^B220^+^ pDC (B) subsets. Histogram plots show the results of CD40, CD86 and H-2K^d^ staining for one mouse out of two in one experiment representative of four independent experiments. Grey shading indicates isotypic controls. For each marker, graphs represent the relative fluorescence intensity (RFI) of individual mice in two independent experiments (n = 3 mice for miR-29b and siRNA9.1, n = 4 mice for miR-127), and are representative of two other independent experiments. *P<0.05 (Mann-Whitney).

To characterize the cellular effectors of miR-29b-induced activation, the phenotype of different subsets of splenic immune cells was assessed *in vivo*, eighteen hours after miRNA systemic delivery to BALB/c mice (Fig. 4). In the mDC CD11c^+^CD11b^+^B220^−^ population (Fig. 4A), miR-29b injection induced the up-regulation of CD40 and CD86 (B7-2) activation markers, as well as of the MHC class I molecule H-2Kd, compared to miR-127 and siRNA9.1-injected mice (p<0.05). The up-regulation of these markers is in line with pro-inflammatory cytokine profiles obtained after *in vitro* treatment of bmDCs (Fig. 1). In the pDC CD11c^low^CD11b^−^B220^+^ population (Fig. 4B), the CD40 and CD86 markers were also significantly up-regulated after miR-29b injection (p<0.05). In our hands, a significant up-regulation of H-2Kd was observed when comparing miR-29b with miR-127. Likewise, the CD3^−^CD49b^+^ NK cells as well as the CD3^+^CD8^+^ and CD3^+^CD4^+^ T-cell populations express the early activation marker CD69 (S4 in File S1). These results demonstrate that injection of miR-29b leads to maturation of antigen-presenting and effector cells.

### Pre-treatment of effector CD8^+^ T-cells with miR-29b before adoptive transfer does not change disease incidence

A direct effect of miR-29b on effector CD8^+^ T-cells was explored using a pre-treatment with miR-29b *in vitro* prior to transfer to Ins-HA mice (S5 in File S1). A disease incidence of 100% was observed for all recipient mice regardless of the number of effector CD8^+^ T-cells transferred. This result suggests the existence of intermediary cellular effectors operative in the protective effect of miR-29b, in line with the results compiled from *in vitro* bmDC experiments, IFNa levels in serum (Fig. 1), and preliminary results from *in vivo* pDC-depletion experiment (S2 in File S1).

### Endogenous miR-29b released in beta cell exosomes elicits immune responses *in vitro*

Finally, we evaluated if natural beta-cell miR-29b shuttled in exosomes could impact immune responses. Exosomes shed by murine MIN6 insulin-secreting beta cells are known to transport auto-antigens such as the Glutamate decarboxylase and to stimulate cytokine secretion by auto-reactive splenocytes from NOD mice, a privileged model of T1D [31], [32]. In addition, exosomes transport mRNA and miRNAs between cells and promote immune activation in acceptor cells (reviewed in [33]). MiR-29b has recently been detected in extra-vesicles released by human pancreatic islets [34].

To investigate whether beta cell derived exosomes contain miR-29b, we generated exosomes from MIN6 culture supernatants using the standard ultracentrifugation method [18]. Nanotracking analysis of MIN6 exosomes showed a size with a mode of 97.0±2.8 nm consistent with the size expected for exosomes (S6A in File S1). Exosome preparations are slightly polydisperse as shown by the presence of minor peaks two- to four-fold bigger in size that might be due to aggregation linked to the method of isolation. Average yields obtained are 6×10^8^ exosomes/ml of MIN6 culture supernatant with purity greater than 5×10^9^ particles/µg of protein. Separation of exosomal proteins by automated electrophoresis reveals a pattern of bands different from MIN6 whole cell lysates consistent with an exosomal protein composition different from that of the original cell (S6B in File S1). The presence of beta cell miRNAs i.e. miR-375, miR-29b, and miR-7a in MIN6 exosomes was confirmed by RT-qPCR (S6C in File S1).

**Figure 5:**
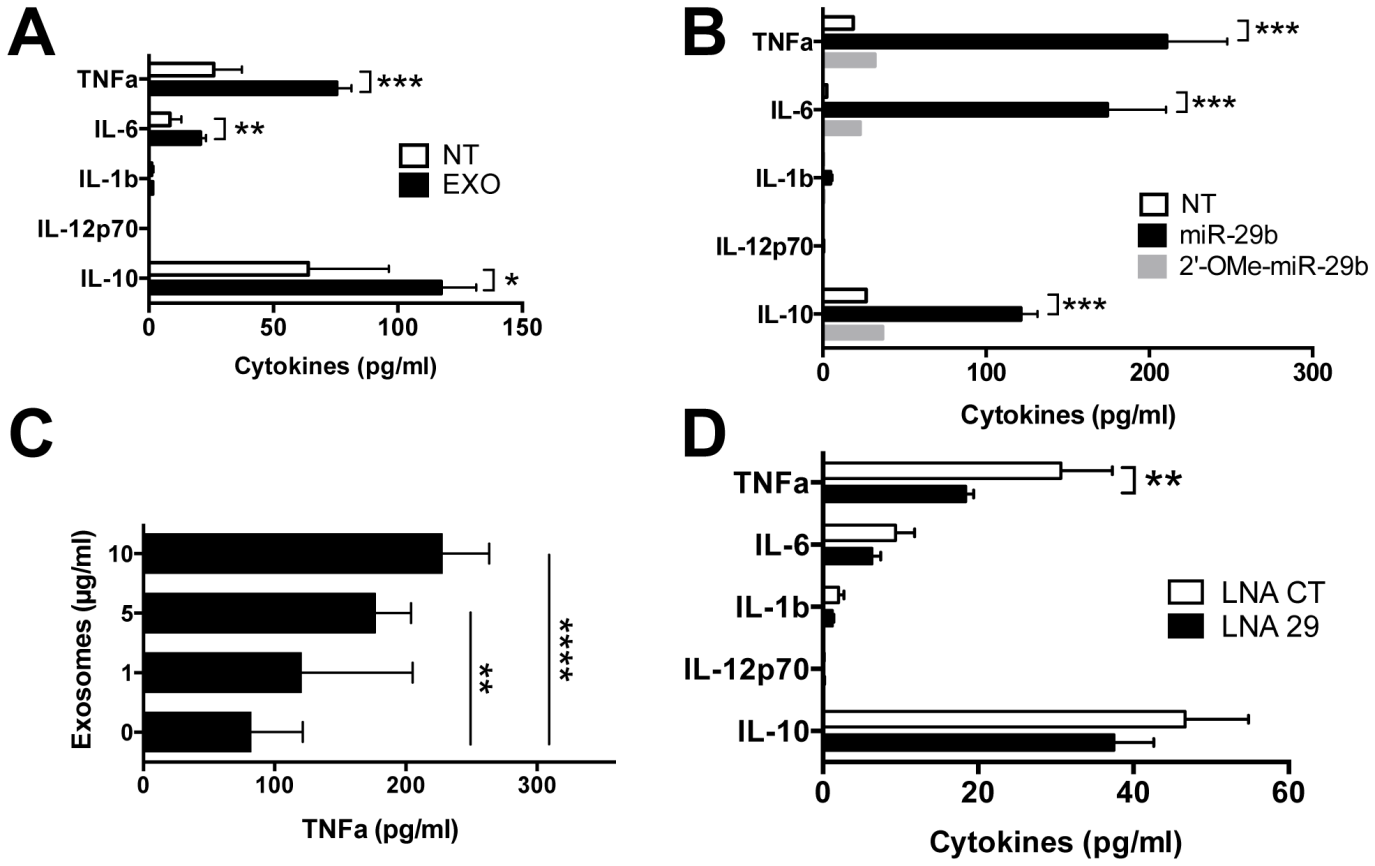
Stimulation of immune cells with exosomes *in vitro*. (A–B, D) Cytokine concentration measured by cytometric bead analysis in supernatants from splenocytes of NOD mice at 48 h of culture (A) with 20 µg/ml of exosomes with n = 7 (NT) and n = 10 (EXO) samples per group from two independent experiments. *P<0.05, **P<0.01 and ***P<0.001 (Mann-Whitney) (B) after transfection with 750 nM of miR-29b or 2′-OMe-miR-29b. Data are representative of two independent experiments (n = 5–6 mice per group). ***P<0.001 (Kruskal Wallis) (C) TNFa concentration in supernatants of RAW264.7 macrophages stimulated for 48 h with various concentrations of MIN6 exosomes. Results from TNFa ELISA analysis are representative of four independent experiments (n = 12 to 15 wells per group). **P<0.001 and ****P<0.0001(Kruskal-Wallis) (D) treatment with exosomes transfected with LNA-miR-29 family inhibitor or control (CT). Data were obtained from n = 7–8 replicates from two independent experiments. **P<0.01 (Mann-Whitney). All bar graphs are presented as mean ± SEM. NT: no treatment.

In downstream immune assays, MIN6 exosomes triggered TNFa, IL-6 and IL-10 secretion from primary cultures of NOD splenocytes (p<0.001, p<0.01, p<0.05 respectively), but no release of IL-12 and IL-1b (Fig. 5A) comparable to the cytokine pattern observed after transfection with the miR-29b analogue (p<0.001, Fig. 5B). In RAW264.7 macrophages, exosome-induced TNFa secretion is dose-dependent (p<0.01 and p<0.0001 at the concentration of 10 and 20 µg/ml respectively, Fig. 5C), recalling dose-responses observed for the miR-29b analogue (S1 in File S1).

To determine whether exosomal miR-29b is engaged in the stimulation of cytokine secretion of NOD immune cells, MIN6 exosomes were transfected with a LNA-miR-29 family inhibitor. A significant drop in TNFa secretion by NOD spleen cells treated with miR-29b knockdown exosomes compared to controls (p<0.01, Fig. 5D) was observed.

## Discussion

Short RNAs trigger innate and downstream adaptive immune responses [22]. Very recently, it has been shown that self miRNAs also interact with receptors of innate immunity, namely TLR-7: in this way, miR-let-7b from cerebrospinal fluids exacerbates neurodegeneration in Alzheimer’s disease [4] and tumour-secreted miR-21 and miR-29a promote prometastatic and inflammatory responses [5]. On the contrary, miRNA administration can also protect mice against tumour development in a TLR-1 NK-cell dependent manner, suggesting that immune signalling pathways might be cell type- or context-dependent [6]. Using miRNA analogues, our study provides evidence that certain beta-cell miRNA sequences efficiently stimulate the TLR-7 receptor in the endosomal compartment. Consistently, miRNA stimulation leads to the secretion of proinflammatory and suppressive cytokines *in vitro* and *in vivo*. We describe here that miR-29b exerts dose-dependent immune modulatory effects, in contrast with other miRNA sequences, arguing in favour of a sequence-dependent mechanism. 2′-O-methyl-ribose modification, a widely used means to hinder receptor-ligand interactions [26], nearly completely abolishes cytokine secretion in the RAW264.7 cell line. Since 2′-O-methyl residues were introduced in the reverse strand, maintaining the guide strand’s integrity, the observed drop in cytokine secretion is clearly independent of the RNAi machinery. Using the TLR-7 antagonist IRS661 [28] or chloroquine to impair TLR activation in the endosome, we show that miR-29b sensing involves the TLR-7 pathway. TLR-2, TLR-3, TLR-4, and TLR-7 stimulation by cognate ligands prevents T1D in the NOD mouse when administered intraperitoneally early in disease development or simultaneously to diabetogenic T-cell transfer [35], [36]. Conversely, TLR-7 stimulation in NOD mice by subcutaneous or topical administration of the ligands CL097 or imiquimod accelerate T1D development [28]. Repeated injections of IRS661 delayed T1D onset, along with a decrease in IFNa levels in the PLNs of prediabetic NOD mice. In this context, our description of miR-29b acting as a TLR-7 ligand raises the question of the putative role of beta-cell miRNAs in the initiation and progression of T1D.

Several studies have reported that extracellular miRNAs are protected from degradation in biological fluids through inclusion in small membrane vesicles of exocytic origin such as exosomes [37], [38] and exosomes are important regulators of immune responses (reviewed in [33]). *In vitro* generated beta cell exosomes transporting beta cell autoantigens have been previoulsy shown to stimulate IFNg, TNFa and IL-6 cytokine production by splenocytes and to activate autoreactive T cells from prediabetic NOD mouse [31]. Subsequently, the author’s identified B lymphocytes and MyD88− dependent TLR-signalling as the major contributors of exosome-mediated immune stimulation [32]. With the aim to evaluate the contribution of endogenous beta-cell miRNAs in an autoimmune context, we tested beta-cell exosomes on spleen cells from NOD mice. As described by Sheng *et al.,* MIN6 exosome preparations induced IFNg (data not shown), TNFa, IL-6, and IL-10 cytokine secretion. Using a LNA miR-29 antagonist, we show that miR-29 molecules shuttled in MIN6 exosomes are immunologically active and significantly weigh on the induction of TNFa secretion in NOD spleen cells.

In line with the assumption that the kinetics of cytokine secretions determine the outcome of immune responses, TNFa contributes to the modulation of autoimmunity leading to type 1 diabetes. TNFa is associated with the beta cell aggression during the early steps of autoimmune diabetes in rodents, but prevents the development of self-reactive T-cells in adult mice [39], [40]. TNFa was detected *in vitro* following miR-29b stimulation of bmDCs and RAW264.7 cells, and MIN6 exosome treatment of NOD spleen cells, and may be implicated in the delayed disease onset observed in our mouse model. Induction of IL-10 secretion by bmDCs in our experiments fits with the overall immunosuppressive effect observed after systemic miR-29b treatment. However, IL-10 secretion by NOD splenocytes does not significantly diminish after LNA-miR-29 inhibition in exosomes, suggesting either a miR-29b independent mechanism, delayed kinetics or masking by the complex exosomal composition.

*In vivo*, we provide evidence that miR-29b indirectly weighs on effectors of adaptive immunity. In a murine model of adoptive transfer of diabetes mediated by antigen-specific CTLs, we show that synthetic miR-29b systemic delivery prevents disease onset. In accordance, insulitis seems less invasive in miR-29b recipient mice, although differences in the homing of CD8^+^ T-cells to the PLNs do not reach statistical significance. Rather, analysis of spleens of recipient mice shows a significant reduction in the number of donor Thy1.1^+^CD8^+^ T-cells, providing a plausible explanation for the reduced cytolytic activity of CD8^+^ T-cells. To our knowledge, this is the first observation of a miRNA sequence modulating an on-going autoimmune response *in vivo*.

MiR-29b parenteral administration is accompanied by an increase in serum IFNa, in parallel to the up-regulation of co-stimulatory molecules (CD40, CD86) and MHC class I molecules (H-2Kd) on conventional mDCs and pDCs. CD11c^+^CD11b^+^ mDCs are operative in peripheral tolerance mechanisms following antigen-presentation, but they are also associated with T-cell priming and activation of diabetogenic responses in PLNs [41], [42]. Like for TNFa, a protective or aggravating role of IFNa/b at different stages of the autoimmune process has been observed [43]. Previous data show that IFNa secretion in response to TLR-7/8 stimulation by nucleic acids is largely pDC-dependent [43], [44]. In a preliminary experiment, administration of a pDC-depleting antibody before miR-29b injection abrogates IFNa secretion *in vivo*, consistent with a contribution of pDCs.

Although miR-29b leads to an up-regulation of the early activation marker CD69 in splenic CD4^+^ and CD8^+^ T-cells in BALB/c mice *in vivo*, a direct impact of miR-29b on transferred CD8^+^ lymphocytes seems unlikely because miR-29b is administered eighteen hours before T-cells and has probably reached target cells before injection of effector T-cells. Also, CD8^+^ T-cells are not easily transfected by RNA-DOTAP liposomes [45] and, though naïve CL4^−^TCR CD8^+^ T-lymphocytes express TLR-7, TLR-7 messenger RNA is repressed in CTLs in our experimental conditions (data not shown). In support of this idea, *in vitro* pre-treatment of CD8^+^ T-cells with miR-29b does not alter disease incidence following transfer *in vivo*.

The endogenous miR-29b, one of the three isoforms of the miR-29 family, is expressed at high levels in pancreatic islet cells [24]. Among identified physiological functions of miR-29b figure proapoptotic regulation of cellular homeostasis, suppression of immune responses to intracellular pathogens [46], [47] or silencing of the beta cell specific monocarboxylate transporter 1 possibly involved in insulin secretion [24]. During the first phases of autoimmune diabetes in NOD mice, miR-29b expression increases with age and immune cell infiltration in islet cells, contributing to beta cell apoptosis by targeting the antiapoptotic protein Mcl1 [25]. In human, the level of circulating miR-29b is increased in children with newly diagnosed T1D [12].

Our hypothesis is that beta-cell miRNAs like miR-29b impact autoimmune responses by recruiting innate immune cells through receptor-ligand interactions, in addition to their important regulatory role. Presumably, injured beta-cell release exosomes loaded with miRNAs and auto-antigens into the extracellular space that may prime resident immune cells and promote expansion of diabetogenic T-cells. On the other hand, studies on mesenchymal stem cell-derived extracellular vesicles revealed their aptitude to inhibit pro-inflammatory islet antigen-specific T-cell responses [48]. Currently, it is difficult to evaluate the physiological relevance of activation of innate immune responses by endogenous miRNAs in the natural history of T1D. However, the absence of miRNA expression in pancreatic beta cells aggravates low dose streptozotocin-induced diabetes in transgenic knock-out mice [49]. Like miR-29b, other endogenous miRNA sequences activating TLR-signalling may provide new insights into the mechanisms underlying inflammatory and autoimmune conditions opening the way for new applications for miRNA mimics in immune-interventions.

## Supporting Information

File S1 **Supporting figures and tables.**
(DOC)
