# Supplementary material for: MicroRNA-29b Modulates Innate and Antigen-Specific Immune Responses in Mouse Models of Autoimmunity
Source: PLoS One. 2014 Sep 9;9(9):e106153. doi: 10.1371/journal.pone.0106153 (PMC4159199; doi:10.1371/journal.pone.0106153)
Supplement: File S1 — Supporting figures and tables. (DOC) [file pone.0106153.s001.doc]

**SUPPORTING INFORMATION**

**S1. Stimulation of TNFa secretion in RAW264.7 cells by miRNA analogues is dose-dependent**.

(A) Dose-dependent stimulation. RAW264.7 cells were plated for four hours before *in vitro* treatment with miR-29b, miR-7a or miR-127 at the indicated concentrations (nM), or were left untreated (NT). After eighteen hours, TNFa production was evaluated in supernatants using an ELISA assay. Results are presented as mean cytokine concentration (pg/ml) ± SEM of three experiments. **P*<0.05, ***P*<0.01 and ****P*<0.001 when compared to untreated cells (Kruskal-Wallis).

(B) Stimulation by double-stranded or single-stranded miR-29b analogue. The miR-29b analogue (750nM) was tested either as double-stranded molecules (duplex F/R) or as single-stranded sequences (F or R). Results are represented as individual values of cytokine concentrations (pg/ml). Data from one representative experiment out of three is shown. **P*<0.05 (Mann-Whitney).


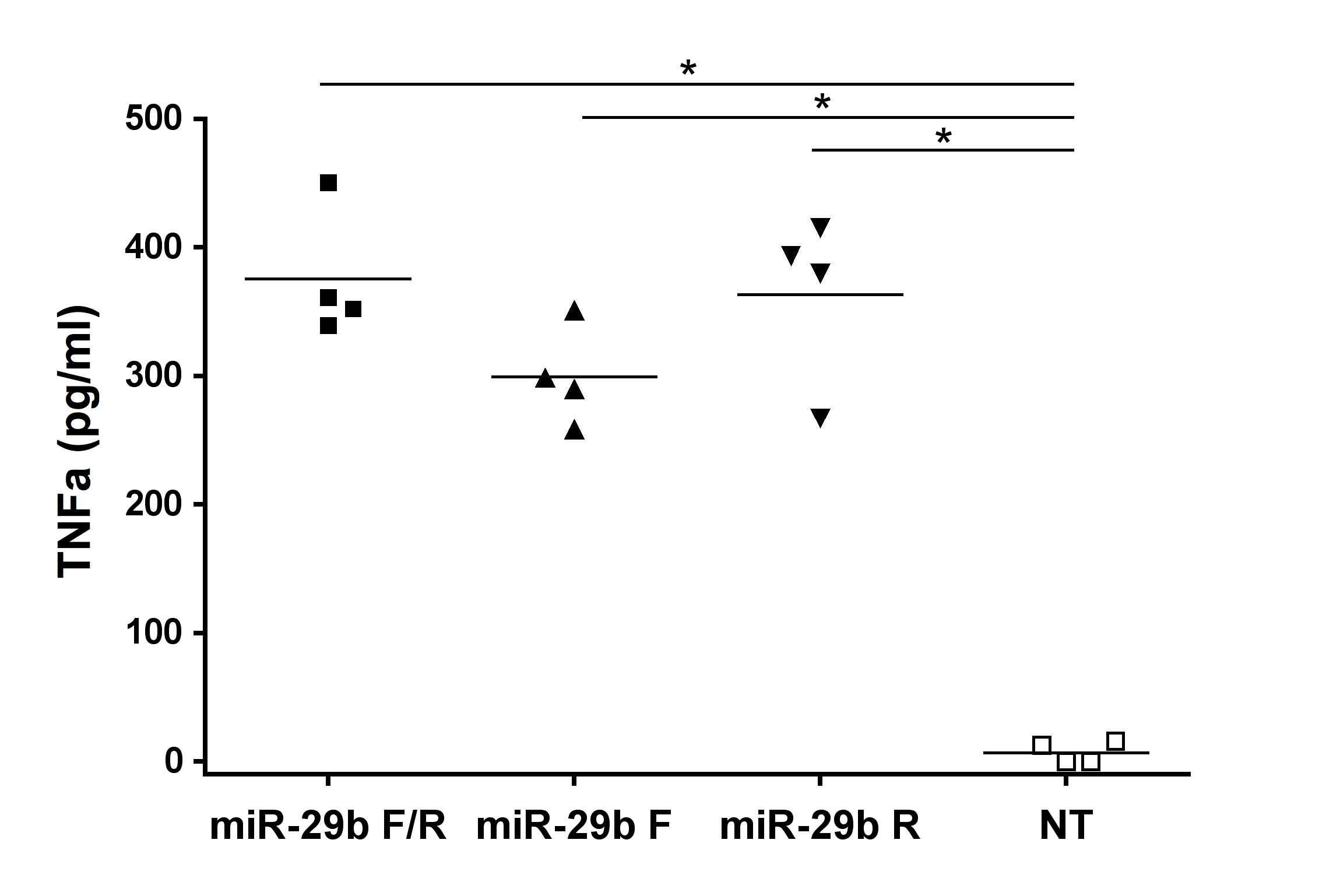

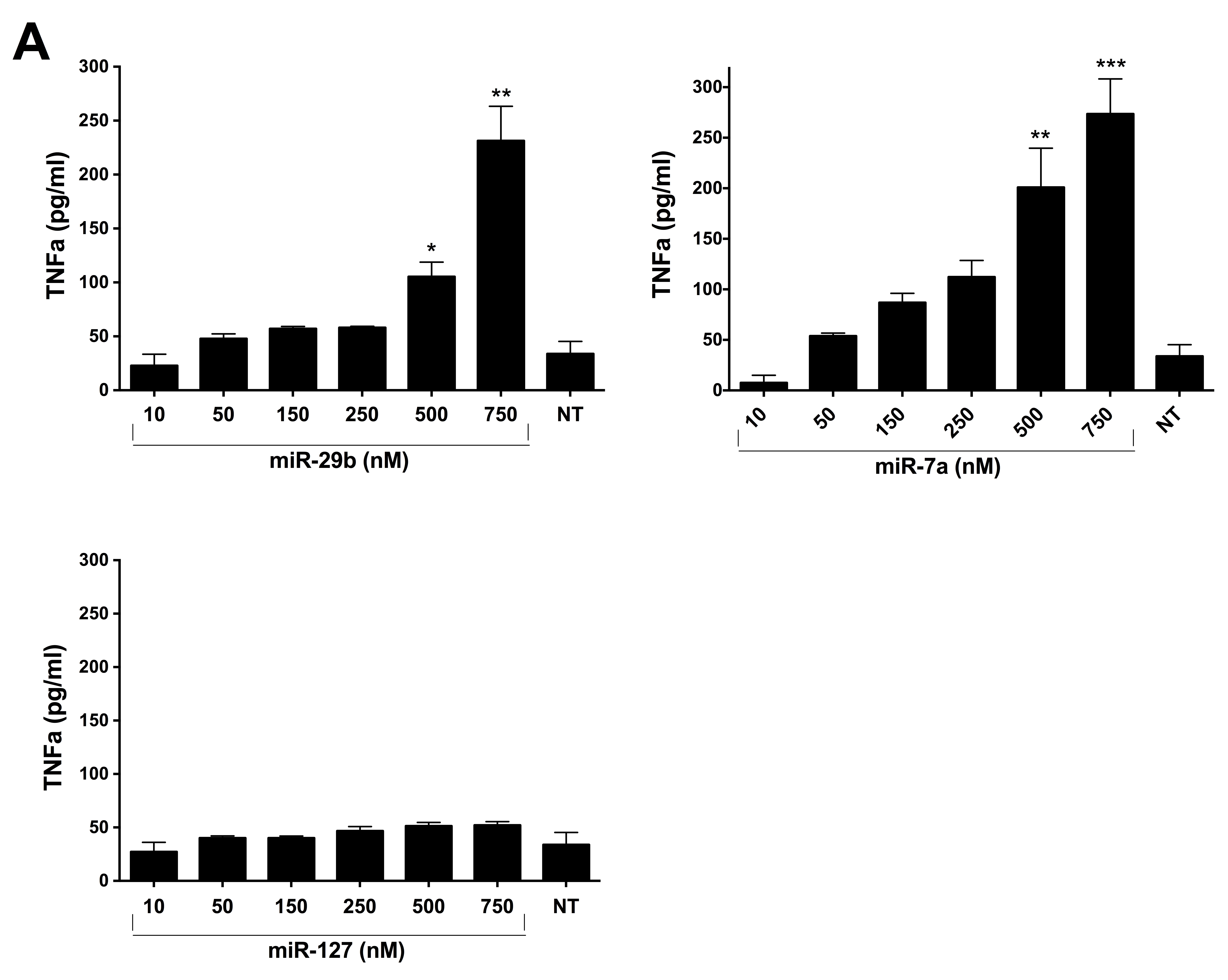


**B**

**S2. IFNa content in the serum of pDC-depleted mice following miR-29b intravenous administration.**

pDCs were depleted *in vivo* in Balb/c mice by the intravenous injection of 500 µg/mouse of the antibody anti-mPDCA-1 functional grade (Miltenyi), 24 hours before receiving miR-29b or the injection buffer HBS. pDC depletion was confirmed by flow cytometry. Serum IFNa was quantified by ELISA seven hours after stimulation. Results are presented as mean concentration of duplicates (pg/ml) ± SEM.


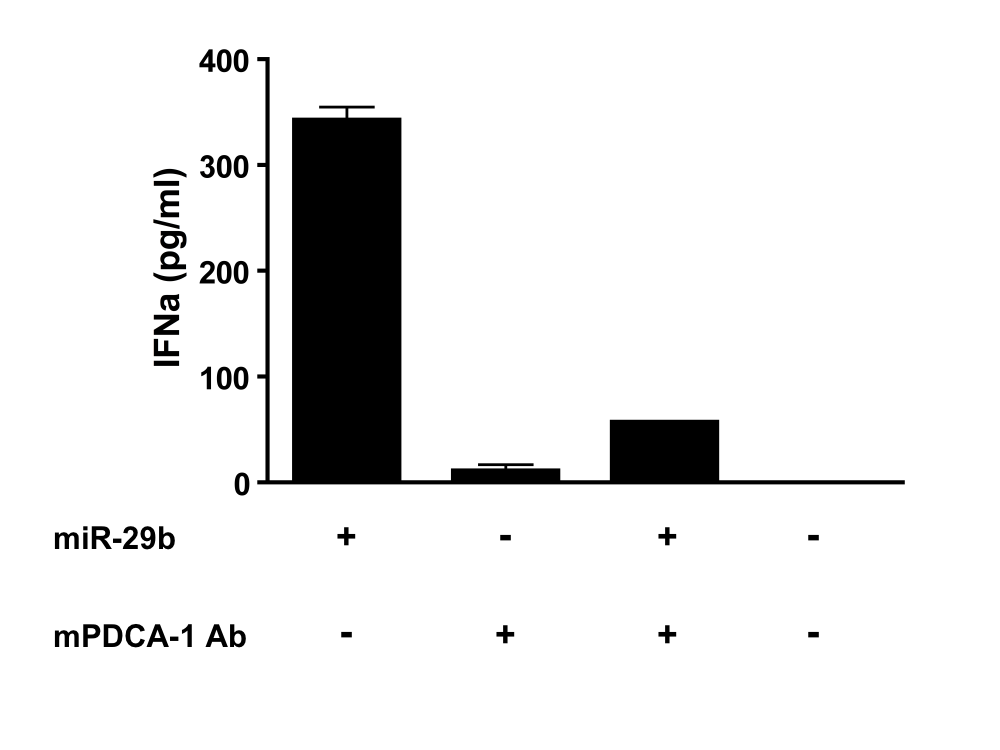


**S3. RNA interference activity exerted by miRNA analogues.**

Experimental evidence shows that the mcl-1 transcript is a natural target of miR-29b [1]. With the aim to investigate RNAi activity of miR-29b analogues, mcl-1 expression was monitored in RAW264.7 macrophages upon transfection with Viromer blue according to manufacturer’s protocol (Lipocalyx, Halle, Germany). Twenty-four hours later, total RNA was extracted from cells using the mirVana RNA extraction kit (Life Technologies), quantified on a Nanodrop Spectrophotometer (Labtech, Palaiseau, France) and controlled for RNA integrity by automated electrophoresis analysis (Experion, Bio-RAD). For the quantification of messenger RNAs, 1 µg of total RNA were treated with TurboDNase (Life Technologies) and reverse transcribed using M-MLV Reverse Transcriptase (Life Technologies) and random 15-mer primers (Eurogentec). Relative standard curve quantification was carried out on an ABI7300 instrument (Applied Biosystems, Life Technologies) using Solisbiodyne reagents (Tarty, Estonie) and primer pair mcl1 (aacgggactggcttgtcaaa/ctgatgccgccttctaggtc) and beta-actin (ttgctgacaggatgcagaag/gtacttgcgctcaggaggag) purchased from Eurogentec. The scatter plot shows b-actin normalized quantities of mcl-1 from two independent experiments. ***P<*0.01 (Kruskal-Wallis).

**S4. Splenic NK, CD8+ and CD4+ T cell activation by miR-29b.**

The expression of the early activation marker CD69 was assessed in splenic NK cells CD3^-^CD49b^+^, CD3^+^CD8^+^ T cells and CD3^+^CD4^+^ T cells following miR-29b, miR-127, or siRNA9.1 intravenous injection in BALB/c mice. Spleens were harvested eighteen hours after injection and CD69 expression was evaluated by flow cytometry. Histogram plots show the results of CD69 staining for one mouse out of two in one experiment representative of three independent experiments. Grey shading indicates isotypic controls.


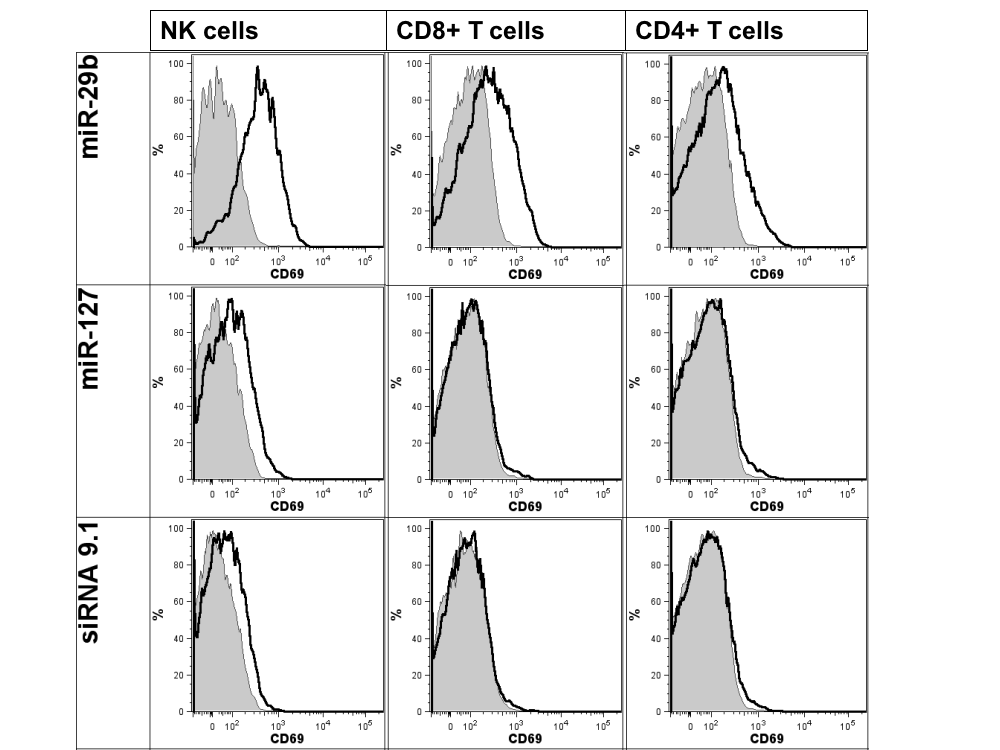


**S5. Adoptive transfer of HA-specific CD8^+^ T-cells pre-treated *in vitro* by miR-29b.**

|  | **Experiment 1**  (diabetic mice/total number of mice) | **Experiment 2**  (diabetic mice/total number  of mice) | |
| --- | --- | --- | --- |
| Number of transferred CD8^+^ T-cells | 5 x 10^5^ | 5 x 10^5^ | 3 x 10^5^ |
| miR-29b | 2/2 | 3/3 | 3/3 |
| siRNA9.2 | 2/2 | 3/3 | 3/3 |
| Untreated | 2/2 | 3/3 | 3/3 |

Two experiments testing miR-29b pre-treatment *in vitro* on HA-specific CTLs were performed in different conditions: experiment 1 was based on the conditions used for bmDC treatment *in vitro* (15 x 10^4^ cells/cm^2^ with miR-29b or control at a final concentration of 150nM); experiment 2 was based on a concentrated T-cell culture (75 x 10^4^ cells/cm^2^ with miR-29b or control at a final concentration of 150nM). After eighteen hours of culture, 3 or 5 x 10^5^ of pre-treated HA-specific CTLs were transferred intravenously to Ins-HA recipient mice. Recipients were monitored for diabetes development. For each situation, the table indicates the number of diabetic mice out of all mice in the group.

**S6. MIN6 cells produce exosomes containing miR-29b**

Exosomes were isolated from MIN6 culture supernatants by differential centrifugation [2] and characterized by (A) nanotracking (NS300-HF488 software NTA3.0, Nanosight, Malvern, Amesbury, UK) and protein analysis using the Bradford assay (Fisher Scientific, Illkirch, France) and (B) Experion Pro260 protein chips (Bio-Rad, Marnes-La Coquette, France). (C) For the detection of exosomal miRNAs, 5µl of exosome suspension were denatured by heating to 70°C for 5 min. and processed for reverse transcription without further purification. Quantification of miRNAs was carried out using RT-stem-loop primers and TaqMan assays (Life Technologies). A ten cycle pre-amplification step was included prior to real-time PCR quantification on an ABI7300 instrument (Life Technologies) using Solis BioDyne reagents (Tartu, Estonie).

1. Roggli E, Gattesco S, Caille D, Briet C, Boitard C, et al. (2012) Changes in MicroRNA Expression Contribute to Pancreatic beta-Cell Dysfunction in Prediabetic NOD Mice. Diabetes 61: 1742-1751.

2. Théry C, Amigorena S, Raposo G, Clayton A (2006) Isolation and characterization of exosomes from cell culture supernatants and biological fluids. Curr Protoc Cell Biol Chapter 3: Unit 3.22.
